# A Plea for Extraction and Replantation as a Means of Cure in Obstinate Alveolar Abscess

**Published:** 1893-04

**Authors:** John S. Marshall

**Affiliations:** Chicago, Ill.


### A Plea for Extraction and Replantation as a Means of Cure in Obstinate Alveolar Abscess.

BY JOHN S. MARSHALL, M.D., OF CHICAGO, ILL.

Read in the Section of Oral and Dental Surgery, at the Forty-third Annual Meeting of the American Medical Association, held at Detroit, Mich., June, 1892.

*Mr. Chairman and Gentlemen of the Section:* —In the presentation of this subject I make no claim to a new discovery, for the operation is an old one and has been occasionally practiced with varying success for many years. I trust, therefore, that I shall be pardoned for introducing a subject in this presence which cannot be classed as a new operation, or an entirely new method of treatment. Methods, however, are sometimes improved, and I shall be content to leave to your good judgment whether the line of treatment to be presently indicated is rational and justifiable, and any improvement over the methods generally pursued.

One of the first and most important principles of surgery is the conservation of diseased organs and tissues, provided they can be restored to a healthy condition, rendered more or less useful for the purposes for which they were created, and incapable of harm to other members or the general system.

In these days of antiseptic surgery, many things are accomplished which a few years ago would have been considered highly dangerous or impossible. The abdominal cavity is now opened with impunity, and operations made upon the enclosed vicera, that to say the least, are startling; the chest is explored and portions of the lung removed; the cranium is perforated, and tumors extracted from the superficial and deeper portions of the brain; and yet the individuals operated upon live to tell the tale.

In no department of minor surgery has greater advancement been made than in dentistry. Most of the diseases of the mouth and associated parts are now more successfully managed, and thousands of diseased teeth are restored to health and usefulness every year, that were formerly consigned to the forceps, and this has been largely the result of a better knowledge of the causation of many diseases, and how to prevent and to combat the inflammatory processes by aseptic methods.

I was led to adopt the practice of extracting and replanting teeth for the cure of obstinate or persistent alveolar abscess on account of the failure—in my own hands and those of others—to cure a considerable percentage of these cases by the ordinary means at our command.

By obstinate or persistent alveolar abscess, I mean those cases of alveolitis in which the inflammatory symptoms persist after all the ordinary means of disinfecting and filling the root canals, etc., have been faithfully tried without avail.

This form of alveolar abscess is usually the result of a crooked root or an abnormally small root canal, making it impossible in either case to remove the putrescent pulp or render innocuous the retained septic material; or perforations of the root made in attempts to open small root canals; or erosion of the apical end of the root; or the presence of broaches, filling material or dressings, which have passed the apical foramen; it is accompanied by a more or less persistent discharge of pus, either through the external or internal plate of the aveolar process, or through the alveolus at the neck of the tooth. Occasionally cases will be found in which no discharges are present, but instead, a chronic induration of the surrounding tissues, or as in the superior teeth, the discharges may find their way into the antrum of Highmore or the anterior nasal passages and possibly mislead the surgeon.

These teeth are usually sooner or later condemned as harmful or worthless members of the body and are removed.

It is this class of teeth for which I make my plea, and I believe from a practical experience extending over a period of several years with this method of treatment, that a majority of them may be rendered healthy and useful for an indefinite period.

It is perhaps hardly necessary to say that replantation is only admissible in the anterior teeth, including the bicuspids. The molars are not capable of being replanted only in exceptional cases, when there is fusion of the roots, and they assume a conical form, and occasionally a lower molar, where the roots are perpendicular to the crown.

I think it will be generally conceded that attempts to cure *in situ* such cases as those just mentioned, usually prove unsuccessful, and that eventually the teeth are lost. This, I believe, results from the fact that such operations are largely, *per force*, only guess work. If the root is curved at a more or less acute angle, it is difficult to follow the canal with the broach, and many times quite impossible; or if the canal is abnormally small, the finest broach may not enter at all. Drilling is unsafe; and under these conditions the various antiseptics and liquified filling materials are unsatisfactory; because they do not always penetrate to the end of the canal, and consequently septic material remains in the root and keeps up a constant irritation.

In cases of erosion of the apical end of the root, amputation of this portion *in situ* is rarely successful in curing the disease, owing to the difficulties in smoothing the stump and perfectly filling the apical foramen. The same may be said of attempts to plug perforations of the sides of the root. In those cases where broaches, filling material or dressings have passed the apical foramen, diagnosis is difficult, except by trephining the alveolar plate and this has no advantage over extraction and replantation.

Roughened surfaces and foreign substances are not kindly borne by the tissues which surround the roots of the teeth. It is therefore imperative that all such hindrances to a return to the normal condition be reduced to a minimum.

For these reasons it would seem preferable to extract and replant such teeth if they do not speedily prove amenable to treatment by the usual methods; for, with the tooth in hand, the root can be minutely inspected and any eroded portion amputated; and the surfaces finely polished. The root canal can be reamed out and cleansed without fear of perforating its sides; the canal and the apical foramen or a perforation, plugged with gold and carefully finished, and the whole thoroughly sterilized by immersion in bichoride of mercury solution 1 to 500 of water (tepid) for thirty minutes.

None of these operations are possible with the same degree of perfection while the tooth is *in situ*; they must be more or less imperfect; and just in that degree will they produce irritation, and the more serious inflammatory processes.

The question might be very properly asked, "Are the operations of replantation and transplantation of freshly extracted teeth having the pericementum attached, founded upon physiological law and sound surgical principles?" I answer, yes! Quite as much so as are the operations of skin and bone grafting, and no one condemns these. Union with the tissues with which they are placed in contact is the result of the same vital processes; the surgical conditions are nearly identical in each of them and success is as certain in the one as in the other; provided the same asceptic conditions can be maintained until union is complete. Failure of replanted teeth to unite with their alveoli is much less common than with transplanted and implanted teeth; at least my personal observation bears out this statement. The immediate cause of failure in replanted, transplanted and implanted teeth is usually suppurative inflammation induced either by mobility of the tooth which constantly breaks up the attachment of the plastic exudate; or septic condition of the tooth, or its alveolus at the time of the operation; or inoculation afterwards from a filty condition of the mouth.

The failures which occur later, viz.: after attachment has taken place, are more difficult to understand. In these cases the surfaces of the roots are attacked by the osteoclasts and gradually honey-combed; or masses of tissue are dissolved at various locations, leaving large cavernous excavations with sharp edges; suppuration accompanies or follows the work of the osteoclasts; the tooth becomes loose, and is sooner or later expelled from the jaw as a foreign substance.

In explanation I would venture the opinion that these phenomena are due to irritation induced by a sceptic condition of the dentine, resulting from decomposition of the organic material contained in it, and that in the form of a gas or effluvium it penetrates the cementum, and, coming in contact with the pericementum and surrounding tissues, sets up this retrograde metamorphosis.

There are certain individuals for whom it would not be wise to undertake the operation, viz.: those suffering from general anæmia, tuberculosis, and syphilis. Such people are never good subjects for surgical operations, as their tissues are very irritable. They do not as a rule heal readily, and are prone to suppuration and sloughing, consequently replantation, transplantation and implantation, if performed upon such persons, are likely to prove unsuccessful.

Many of the failures from these operations that have come under my notice, have been associated with one or the other of these diseases. Great care should therefore be exercised in the selection of the cases upon which to operate. Carelessness in this regard can only result in failure.

Of the many operations which it has been my privilege to perform, it has not thus far been my misfortune to be obliged to record a single failure, although they have comprised all grades of the disease, several of long standing, and ranging in duration from a few months to fifteen years. But I refrain from mentioning individual cases, for fear of wearying you.

The after treatment consists in frequent irrigations of the mouth with antiseptic solutions; among the best of which are the Thiersch solution composed of boric acid, 12 parts; salicylic acid, 4 parts; water, 1000 parts; and another consists of listerine, 1 part; water, 3 parts.

Bichloride of mercury solutions are not permissible on account of the danger from poisoning and the blackening of the teeth.

We are all no doubt interested to know the character of the union which takes place between the root of the replanted tooth and its alveolus, but so far it has not been positively demonstrated. I would therefore like to offer a few thoughts which I trust may tend towards the elucidation of this question.

Applying the methods employed in physical diagnosis to these cases, we find that percussion gives the most marked signs. Taking the percussion note of normal teeth, produced by striking the tooth with a steel instrument as the standard of pitch, we find that as inflammatory conditions of the alveolus advance, the percussion note becomes lower and duller; while on the other hand, as these symptoms subside, the note assumes a clearer and higher pitch. This lowering of the tone is doubtless the result of a thickening of the pericemental membrane, and its increased vascularity.

The percussion note given by a large percentage of replanted teeth a few months after the operation or when union is complete, is much clearer and higher pitched than that of the adjoining teeth. This is more noticeable in the superior than the inferior teeth, on account of the greater resonance of the superior maxilla.

These facts would seem to indicate that a bony union takes place in these cases between the root and its alveolus. It would also seem probable that the locations at which this anchylosis would most likely occur, would be where the pericementum had been destroyed, or the cement tissue partially removed, and I can see no reason why under these conditions union may not take place in the same manner as with fractured bones. In other cases the percussion note is normal. This would indicate a normal reunion of the pericementum with the alveolus; but where the percussion note is lower and duller, it would be certain evidence of an indurated pericementum, or other inflammatory symptoms.

In conclusion, let me emphasize the following points as necessary to insure success in the operations :

1. Exclude anæmic, tubercular and syphilitic cases.

2. Secure thorough aseptic conditions of the surfaces, of the root and pulp canal, by washing and immersing in bichloride of mercury solution 1 to 500 of water.

3. Amputate and smooth all eroded surfaces but sacrifice as little of the pericementum as possible. This is very important.

4. Hermetically seal the pulp canal, and apical foramen, and any perforations that may exist, with gold fillings.

5. Curette the abscess cavity, remove the blood clot from the alveolus, and wash both with the bichloride of mercury solution before replanting the tooth.

6. Secure immobility of the tooth by a ligature or an interdental splint, until union has taken place.

